# Supplementary material for: Brace compliance process in adolescents with spinal deformities: A qualitative study
Source: PLoS One. 2024 Aug 2;19(8):e0305754. doi: 10.1371/journal.pone.0305754 (PMC11296642; doi:10.1371/journal.pone.0305754)
Supplement: Attachment — Submitted filename: Responses to Reviewers.docx [file pone.0305754.s002.docx]

Journal Requirements:

When submitting your revision, we need you to address these additional requirements.

1. Please ensure that your manuscript meets PLOS ONE's style requirements, including those for file naming. The PLOS ONE style templates can be found at
   https://journals.plos.org/plosone/s/file?id=wjVg/PLOSOne_formatting_sample_main_body.pdf and 
   <https://journals.plos.org/plosone/s/file?id=ba62/PLOSOne_formatting_sample_title_authors_affiliations.pdf>

**Response.** Many thanks; we rechecked the PLOS ONE style templates and revised some points.

2. We suggest you thoroughly copyedit your manuscript for language usage, spelling, and grammar. If you do not know anyone who can help you do this, you may wish to consider employing a professional scientific editing service.

Whilst you may use any professional scientific editing service of your choice, PLOS has partnered with both American Journal Experts (AJE) and Editage to provide discounted services to PLOS authors. Both organizations have experience helping authors meet PLOS guidelines and can provide language editing, translation, manuscript formatting, and figure formatting to ensure your manuscript meets our submission guidelines. To take advantage of our partnership with AJE, visit the AJE website (http://learn.aje.com/plos/) for a 15% discount off AJE services. To take advantage of our partnership with Editage, visit the Editage website (www.editage.com) and enter referral code PLOSEDIT for a 15% discount off Editage services.  If the PLOS editorial team finds any language issues in text that either AJE or Editage has edited, the service provider will re-edit the text for free.

Upon resubmission, please provide the following: 
● The name of the colleague or the details of the professional service that edited your manuscript
● A copy of your manuscript showing your changes by either highlighting them or using track changes (uploaded as a *supporting information* file)
● A clean copy of the edited manuscript (uploaded as the new *manuscript* file)

**Response.** Thanks for your advice. Hiva Razavi, one of the authors, has thoroughly edited the manuscript. Both *supporting information* and *manuscript* files will be submitted.

3. PLOS requires an ORCID iD for the corresponding author in Editorial Manager on papers submitted after December 6th, 2016. Please ensure that you have an ORCID iD and that it is validated in Editorial Manager. To do this, go to ‘Update my Information’ (in the upper left-hand corner of the main menu), and click on the Fetch/Validate link next to the ORCID field. This will take you to the ORCID site and allow you to create a new iD or authenticate a pre-existing iD in Editorial Manager. Please see the following video for instructions on linking an ORCID iD to your Editorial Manager account: https://www.youtube.com/watch?v=_xcclfuvtxQ

**Response.** As you requested, the corresponding author’s ORCID information was updated on the author's page.

4. We note you have included a table to which you do not refer in the text of your manuscript. Please ensure that you refer to Table 2 in your text; if accepted, production will need this reference to link the reader to the Table.

**Response.** Many thanks for your attention. Table ‘numbers and captions have been revised and checked.

5. Please include captions for your Supporting Information files at the end of your manuscript, and update any in-text citations to match accordingly. Please see our Supporting Information guidelines for more information: http://journals.plos.org/plosone/s/supporting-information.

**Response.** The captions of the Supporting Information files were added at the end of the manuscript.

**Reviewer# 1**: Dear reviewer: thank you for your thoughtful and thorough review of our manuscript.

Dear editor,
Thank you for your kind invitation to review the study entitled "Brace compliance process in adolescents with spinal deformities: a qualitative study".
I congratulate the authors for the effort they put into this study. I think it is a very important study, but there is a significant methodological problem.
Asymmetric braces are effective in the treatment of scoliosis and Milwaukee brace has not been used in the treatment of scoliosis for about 20 - 25 years. In this sense, I strongly recommend the authors to update their information.
“The Milwaukee brace has a negative influence on quality of life, physiological problems, sleep disorders and many disadvantages in the areas of body image. However, the most significant of these is its appearance, which reduces its acceptance due to cosmetic deterioration and discomfort “
It is known that braces that are designed asymmetrically, with spaces where the body can go opposite the corrective zones, are effective.
Ref: Weiss HR, Çolak TK, Lay M, Borysov M. Brace treatment for patients with scoliosis: State of the art. S Afr J Physiother. 2021 Oct 26;77(2):1573. doi: 10.4102/sajp.v77i2.1573. PMID: 34859162; PMCID: PMC8603182.
Weiss, H. R., & Kleban, A. (2015). Development of CAD/CAM Based Brace Models for the Treatment of Patients with Scoliosis-Classification Based Approach versus Finite Element Modelling. Asian spine journal, 9(5), 661–667. https://doi.org/10.4184/asj.2015.9.5.661
Weiss, H. R., Lay, M., Seibel, S., & Kleban, A. (2021). Ist eine Verbesserung der Behandlungssicherheit in der Korsettversorgung von Skoliosepatienten durch Anwendung standardisierter CAD-Algorithmen möglich? [Is it possible to improve treatment safety in the brace treatment of scoliosis patients by using standardized CAD algorithms?]. Der Orthopade, 50(6), 435–445. <https://doi.org/10.1007/s00132-020-04000-9>

- It is also unclear why the researchers used a Milwaukee brace and not a kyphosis brace in patients with kyphosis.

**Response.** Many thanks for highlighting this point. We agree that using modern lower-profile orthoses has better psychosocial outcomes for individuals with AIS or kyphosis. However, in this qualitative study, our aim was not to evaluate the superiority of one orthosis to another. Moreover, the Milwaukee brace is the most prescribed orthosis in our country for no-surgical treatment of adolescents with spine deformities. Although there are some orthotists in our country who can manufacture asymmetric braces, they recently received their certification. Therefore, the number of prescriptions for the Milwaukee brace is still high. To address your valuable comment, we have added some sentences in the limitation section of the manuscript with the supporting references you provided. Regarding kyphosis patients, the Milwaukee brace was prescribed because all the included cases had a large kyphosis curve with an apex of above T8.

- I suggest the authors to redesign their study. In this age group, it is important that they receive appropriate treatment, especially during the period of rapid progression of scoliosis. Although the efficacy levels of scoliosis braces are reported differently in different studies, the most effective braces are asymmetric braces.

**Response.** Many thanks for your attention. You are absolutely right, and we also acknowledge the effectiveness of asymmetric braces based on previous studies. However, the purpose of this study was not to investigate the superiority of one brace to another. In this study, our aim was to investigate the process of brace compliance based on the experiences of adolescents who are using a brace. In this study, the authors did not have any intervention or authority to prescribe and suggest the type of brace, and they only invited interviewees from the three orthotic and prosthetic centers to be included. Since the treating spine surgeons refer to their textbook to prescribe the best orthotic option for adolescents with spine deformities, conventional brace, such as the Milwaukee brace and Boston brace, are our country's most prevalent prescribed orthosis. It should be noted that some orthotists can manufacture the asymmetric braces in our country, and future studies can be designed to evaluate the superiority of asymmetric braces to symmetric braces. Moreover, this will be a fruit area for researchers to assess the lived experiences of adolescents using an asymmetric brace and compare their experiences with those using a symmetric brace. Based on your suggestion, we mentioned this issue as a limitation in the discussion section and suggested conducting studies on adolescents using modern and up-to-date braces. Please see page 24, lines 491-494. This is clarified in the limitation section of the manuscript.

- Furthermore, the authors wrote that cases between 10 and 14 years of age would be included, but the average age presented in the table is older than 14 years. When the standard deviation is added, it is seen that even 17-year-old individuals are included.

**Response.** Thanks for your comment. Actually, all participants were 10-14 years old at the time of deformity diagnosis and starting the brace treatment. We invited those with experience using a brace for at least three months for the interviews. As a result, the average age of the participants during the interviews was more than 14 years. The participants told the interviewer about their past and experiences from the days of deformity diagnosis and the beginning of the brace treatment to the day of the interview. Based on your comment and to clarify this, some corrections were made in the manuscript on Page 5, line 89.

- The authors did not present the Cobb angles of the patients.

**Response.** Many thanks for highlighting this point. As per your suggestion, the mean Cobb angle at the time of referring to the orthotic center and beginning braces treatment was added to Table 3.

- It was reported that children who had just started using a brace would be included in the study, but it was reported that children had been using a brace for more than 8 months.

**Response.** It has been stated that “the study required adolescents who had undergone brace treatment for more than three months.” The participants told the interviewer about their past and experiences from the days of deformity diagnosis and the beginning of the brace treatment to the day of the interview.

- I suggest the authors not to use abbreviations in the abstract.

**Response.** Many thanks for your comment. The suggested changes have been made in the abstract section, and the abbreviations have been eliminated.

Reviewer #2: Dear reviewer: thank you for your thoughtful and thorough review of our manuscript.

The authors present an interesting on a less debated argument. The qualitative approach in considering brace compliance in AIS and SK is very unusual and give interesting and useful information.
In the present form the manuscript is not suitalble for publication and need some clarifications and revisions.

- The paper is too long and of difficult reading.

**Response.** Thanks for your valuable comment. We tried to summarize the text as much as possible. Moreover, some of the quotations have been eliminated.

- The methods and results section are unproportionally long if compared to discussion and introduction

**Response.** Many thanks for highlighting this point. Based on the journal's guidelines, we have designed the method section and reported the results per the [Consolidated criteria for reporting qualitative research (COREQ) checklist](http://intqhc.oxfordjournals.org/content/19/6/349.long).  Considering that all checklist items should have been included in the manuscript, the method and result sections are long. However, based on your comment, we have summarized these sections as much as possible. The participants' quotations have been reduced to reduce the content volume of the results.

- The qualitative approach is main purpose of the study but it could be useful to summarize questions of the review perhaps a table reporting all the question could de add to clarify.

**Response.** Thanks for paying attention to this issue. The guide questions used in the interviews were prepared and submitted as a supplementary file. Based on your opinion, the questions are now in the table 1 and added through the manuscript on pages 3 and 4.

- In reporting the obatained results a more organic approach could be useful perhaps categorising the answers and giving some final picture of the collected results.

**Response.** Many thanks for highlighting this point. Considering that the design of our study was qualitative, the content analysis of the results led to code extraction, which was further reported as sub-categories and categories formation. The text of the interviews was studied, and their results were coded, which led to the creation of classes and concepts. Therefore, changing how the results are reported may not be in line with created categories and sub-categories. However, the results summary is presented graphically in Figure 1 to address your valuable comment.

- Have the authors noted a difference in compliance between the TLSO and Milwaukee users? If yes have they found a clear reason?

**Response.** Thanks for your comment. Since the present study was designed as a qualitative study, no statistical analysis was applied. Therefore, we could not accurately report whether there was any difference between these two braces.

- The discussion section has to unique and no divided in section

**Response.** The suggested changes have been made in the discussion section. In this revised version of the manuscript, the discussion sections have been presented as an integrated part.
